# Professor Dr. Hans-Dieter Klenk (1938–2021)

**DOI:** 10.1080/22221751.2021.1949249

**Published:** 2021-07-15

**Authors:** Stephan Becker, Heinz Feldmann, Jürgen A. Richt, (on behalf of many friends and colleagues)

**Affiliations:** a Institut für Virologie, Philipps-Universität Marburg, Marburg, Germany; b Laboratory of Virology, National Institute of Allergy and Infectious Diseases, National Institutes of Health, Hamilton, MT, USA; c Department of Diagnostic Medicine/Pathobiology, College of Veterinary Medicine, Kansas State University, Manhattan, KS, USA


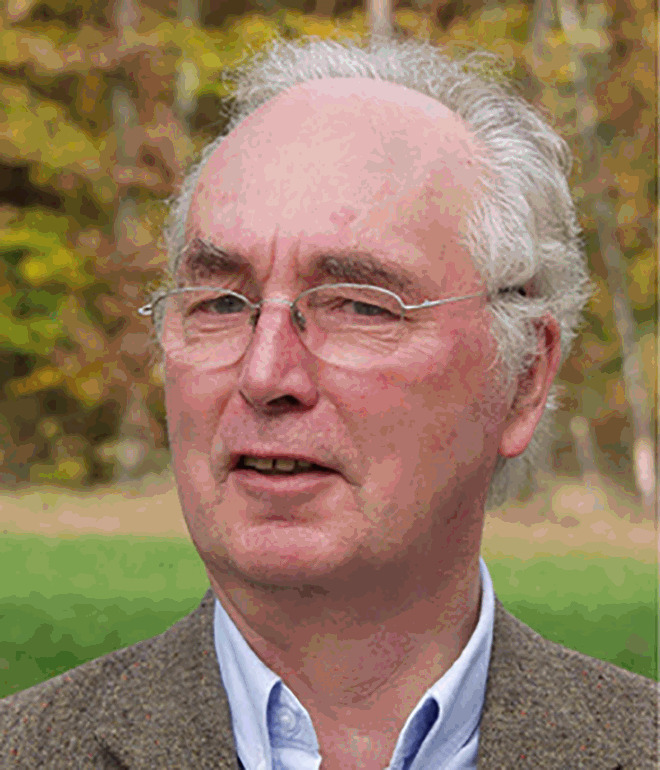


We share with great sadness that our long-time friend and colleague Prof. Dr. Hans-Dieter Klenk passed away on 1 June 2021, at the age of 82. With the passing of Hans-Dieter Klenk, the worldwide virology and infectious disease community is losing one of its most eminent representatives who has shaped the scientific landscape of these disciplines for decades.

Hans-Dieter Klenk studied medicine and biochemistry in Tübingen, Vienna and Cologne. From 1967 to 1970 he started his outstanding scientific career as a visiting scientist in the laboratory of Purnell W. Choppin at the Rockefeller University in New York. In 1973, back in Germany, he was appointed as a Full Professor in Virology at the Justus Liebig University of Gießen, and in 1985 he became the Chair of the Department of Virology at the Philipps University of Marburg. He held this position until his retirement in 2007. It did not come as a surprise to any of his colleagues, friends and family, he continued to serve science in many ways until his death.

Prof. Dr. Klenk has decisively shaped Germany’s virology with a strong international orientation and visibility. Under his leadership, numerous scientists not only had the opportunity to develop their own scientific careers, but were also appointed in leading positions at national and international institutions in academia, industry and government. He used the power of new molecular, genetic, biochemical, and cellular methods to study viruses and identify the functions of their specific genes and proteins. Directly associated with the name Hans-Dieter Klenk is the very successful research work on influenza viruses, in particular the role of the surface protein hemagglutinin in the pathogenesis of influenza infections. In addition, other zoonotic viruses that threaten public health as emerging viruses, such as Ebola, Marburg and Lassa viruses, were of great interest to him. He founded the BSL4 laboratory in Marburg and gave the Department of Virology a completely new infectious disease research direction, which has been highly successful and competitive and is still very actively pursued today.

Prof. Dr. Klenk served as the president of the German Society for Virology from 1999 to 2005. He has been the vice-president of the von Behring-Röntgen-Stiftung, Gießen-Marburg, since its foundation. He coordinated numerous scientific consortia, including the highly successful Collaborative Research Center SFB 286 of the German Research Foundation (DFG), and was active on editorial boards of multiple scientific journals with specific involvement in the establishment of the journal Emerging Microbes & Infections (EMI). He served as an advisory board member for many scientific institutions including but not limited to the Georg-Speyer-Haus, the Feldberg Foundation for Anglo-German Scientific Exchange, the Institute Pasteur of Shanghai, and the Institute of Medical Microbiology, Fudan University. After his retirement, he specifically invested time supporting Chinese virologists in collaboration with leading scientists in China.

Hans-Dieter Klenk was an intellectual leader in his field, in the pantheon of virologists, for many decades. He has received numerous highly respected scientific prizes for his work, among those the Robert Koch Medal, the Ernst Jung Medal for Medicine and the Emil von Behring Prize. In addition, he was awarded the Grand Cross 1st class of the Order of Merit of the Federal Republic of Germany in 2018.

His death weighs heavy on our hearts and minds as we all will miss this outstanding and visionary scientist, this strong and commanding leader, this supportive and respectful mentor and advisor, and this straightforward, humorous, and generous person that he was until the very end.

Our deepest and sincere sympathy goes out to his wife Ulrike, their three sons Christoph, Philipp, Alexander, and the entire extended Klenk family.

Our sincere gratitude to Hans-Dieter Klenk whose science and personality has shaped our careers and lives.

